# Determinants of oral health–related quality of life in older adults receiving maintenance hemodialysis

**DOI:** 10.3389/fpubh.2026.1845708

**Published:** 2026-07-22

**Authors:** Pan Wang, Shan-Shan Hong, Shi-Yun Lu, Yu Wang, Yan-Yu Liu, Xiu-Li Feng, Li-Hua Yang, Jie-Li Chen, Yu Ding, Wei-Feng Chen

**Affiliations:** 1 Blood Purification Centre, The Second Affiliated Hospital, Guangzhou Medical University, Guangzhou, China; 2 Department of Respiratory Medicine, Panyu Branch, The Second Affiliated Hospital, Guangzhou Medical University, Guangzhou, China; 3 Department of Emergency, Panyu Branch, The Second Affiliated Hospital, Guangzhou Medical University, Guangzhou, China; 4 Management Committee, Panyu Branch, The Second Affiliated Hospital, Guangzhou Medical University, Guangzhou, China; 5 Department of Neurology, Panyu Branch, The Second Affiliated Hospital, Guangzhou Medical University, Guangzhou, China; 6 Department of Vascular Surgery, Panyu Branch, The Second Affiliated Hospital, Guangzhou Medical University, Guangzhou, China; 7 Department of Obstetrics and Gynecology, Panyu Branch, The Second Affiliated Hospital, Guangzhou Medical University, Guangzhou, China

**Keywords:** influencing factors, maintenance hemodialysis, older adults, oral health self-efficacy, Oral health-related quality of life

## Abstract

**Objective:**

This study aimed to assess the current status of oral health–related quality of life (OHRQoL) and to identify associated factors among older adults receiving maintenance hemodialysis (MHD).

**Methods:**

Between September 2024 and September 2025, a total of 107 older adults receiving MHD were recruited from a Grade A tertiary hospital in Guangdong Province using a convenience sampling method. Data were collected using a general information questionnaire, the Oral Health Impact Profile-14 (OHIP-14), and the Self-Efficacy Scale for Oral Self-care (SESS). Univariate analyses and stepwise multiple linear regression were performed to determine factors associated with OHRQoL.

**Results:**

The mean OHIP-14 score was 18.97 ± 8.00, and the mean SESS score was 43.52 ± 12.98. A statistically significant negative correlation was observed between OHIP-14 and SESS scores (*r* = −0.736, *p* < 0.01). Multivariate analysis identified dialysis frequency, dialysis duration, tooth brushing frequency, tooth brushing duration, pre-dialysis blood urea nitrogen, pre-dialysis serum creatinine, SESS score, and comorbid diabetes mellitus as independent factors associated with OHRQoL (*p* < 0.05).

**Conclusion:**

Older adults receiving MHD demonstrated relatively low levels of both OHRQoL and oral self-efficacy. Enhanced clinical attention and the development of targeted interventions addressing the identified influencing factors may contribute to improvements in OHRQoL in this population.

## 1 Introduction

Patients receiving maintenance hemodialysis (MHD) frequently experience oral complications secondary to renal failure, including halitosis, xerostomia, and oral mucosal lesions, which may impair masticatory and swallowing functions (1). Previous studies have reported that the prevalence of moderate-to-severe periodontitis, thirst, oral pain, and ammonia-related halitosis among individuals receiving MHD may reach 93.3, 68.9 to 86%, 72, and 53.3%, respectively, which are significantly higher than those observed in the general population (2, 3). These oral health problems are particularly pronounced among older adults receiving MHD and have been shown to exert substantial effects on physical, psychological, and social functioning (4, 5).

The process of aging is associated with physiological atrophy of the salivary glands, diminished manual dexterity, and a higher incidence of polypharmacy (encompassing antihypertensives, anticholinergics, and calcium channel blockers). All these factors exacerbate xerostomia and undermine oral self-care (5, 6). Moreover, cognitive decline may lead to reduced compliance with proper toothbrushing techniques. When combined with end-stage renal disease (ESRD), these age-related alterations impose a distinct and severe burden on oral health. However, the interaction among aging, dialysis-related factors, and oral health remains inadequately investigated.

Oral health-related quality of life (OHRQoL) is a patient-centered outcome measure that evaluates the physical, psychological, and social implications of oral conditions (7). It is commonly assessed using validated instruments such as the Oral Health Impact Profile-14 (OHIP-14). Although OHRQoL has been investigated in general populations with mental health disorders (8), three significant research gaps remain. First, most existing studies have included patients across all age groups. Despite age-related physiological changes that influence oral health, age-stratified data specifically for older adults (aged ≥60 years) are limited. Second, oral self-efficacy—a crucial psychological determinant of health behaviors—has not been sufficiently explored, even though it is known to predict oral hygiene practices and outcomes in other chronic disease populations (9). Third, the independent contribution of objective biochemical markers (e.g., pre-dialysis blood urea nitrogen [BUN] and serum creatinine) to OHRQoL, after accounting for self-efficacy and oral hygiene behaviors, remains unclear. Elevated levels of BUN and creatinine reflect the accumulation of uremic toxins, which can alter oral microecology and lead to ammonia-related halitosis, potentially affecting social interactions and quality of life (1). Furthermore, diabetes—a major comorbidity in patients receiving MHD—has well-established bidirectional associations with periodontitis and systemic inflammation; however, its independent effect on OHRQoL in older MHD patients has not been fully elucidated.

To address these research gaps, the present study was devised to investigate the current status of oral health-related quality of life (OHRQoL) among older adults patients undergoing MHD and to identify the influencing factors. It was hypothesized that: (1) older adults MHD patients present with poor OHRQoL; (2) dialysis frequency, dialysis duration, oral hygiene behaviors (toothbrushing frequency and duration), pre-dialysis blood urea nitrogen (BUN), pre-dialysis creatinine, diabetes mellitus, and oral self-efficacy are independent predictors of OHRQoL. The findings may provide evidence for targeted multidisciplinary interventions intended to improve oral health outcomes in this vulnerable population.

## 2 Participants and methods

### 2.1 Study population

This study employed a convenience sampling method to recruit 107 patients receiving regular hemodialysis at a Grade A tertiary hospital in Guangdong Province between September 2024 and September 2025. Inclusion criteria were as follows: (1) age ≥60 years; (2) duration of hemodialysis ≥3 months (The first 3 months of hemodialysis are characterized by metabolic instability, frequent hospitalizations, and a high burden of uremic symptoms; after 3 months, most patients achieve a relatively stable MHD phase) (10). Including patients with a dialysis duration of less than 3 months might introduce confounding due to the effects of acute illness on oral health (11). Therefore, a minimum dialysis duration of 3 months was chosen as an inclusion criterion, and (3) preserved consciousness with voluntary participation. Exclusion criteria: A confirmed diagnosis of Sjögren’s syndrome or other autoimmune diseases influencing the salivary glands; a history of head-neck radiotherapy; active malignancy necessitating chemotherapy or radiotherapy; severe arthritis or Parkinson’s disease that hindered independent toothbrushing (assessed by the clinician).

### 2.2 Measurement instruments

#### 2.2.1 General information questionnaire

The general information questionnaire was developed by the research team and included demographic characteristics, lifestyle factors, and clinical biochemical indicators (e.g., pre-dialysis blood urea nitrogen, serum creatinine, and C-reactive protein).

#### 2.2.2 Oral health impact Profile-14 scale

The Oral Health Impact Profile-14 (OHIP-14), developed by Slade and Spencer (12) in 1994, was used to assess OHRQoL. The instrument comprises 14 items across seven dimensions and is rated using a 5-point Likert scale, with total scores ranging from 0 to 56; higher scores indicate greater severity of oral health-related impairments. The instrument was translated into Chinese by Xin in 2006 (13). A total score ≥12 indicates poor OHRQoL, whereas a score <12 indicates good OHRQoL. The Chinese version has demonstrated good reliability and validity (Cronbach’s *α* = 0.930).

#### 2.2.3 Self-efficacy scale for oral self-care

The Self-Efficacy Scale for Oral Self-care (SESS), developed by Kakudate et al. (14) in 2007, was subsequently translated and adapted into Chinese by Xin and Lin (13). The Chinese version has demonstrated good reliability and validity (Cronbach’s *α* = 0.879) and is suitable for assessing oral self-efficacy (15). The scale comprises 15 items across three dimensions—regular dental check-ups, appropriate toothbrushing, and a balanced diet—and is rated using a 5-point Likert scale ranging from “not confident at all” to “very confident,” with scores assigned from 1 to 5. Total scores range from 15 to 75, with scores of 15 to 53 indicating low self-efficacy, 54 to 59 indicating moderate self-efficacy, and 60 to 75 indicating high self-efficacy (8).

### 2.3 Data collection

Data collection was conducted collaboratively by nursing staff from the hemodialysis unit and the department of dentistry using a face-to-face questionnaire administration approach. Prior to data collection, investigators provided a detailed explanation of the study objectives and instructions for questionnaire completion. All questionnaires were completed anonymously after informed consent had been obtained from all participants. A post-hoc power analysis was carried out via G*Power 3.1. In the context of multiple linear regression involving up to 11 independent variables, with an effect size of *f*^2^ = 0.35 (medium-to-large), a significance level (*α*) of 0.05, and a statistical power of 0.80, the requisite sample size was computed to be 55. The sample size of 107 in our study surpassed this calculated value, suggesting sufficient statistical power. However, the sample size is relatively small, and the results ought to be interpreted with prudence, especially when conducting subgroup analyses. A total of 115 questionnaires were distributed, of which 110 were returned, and 107 were deemed valid, resulting in an effective response rate of 93.0%.

### 2.4 Quality control

(1) Prior to data collection, all personnel involved received standardized training to ensure a comprehensive understanding of the study objectives and were responsible for guiding and supervising participants during questionnaire completion. (2) All questionnaire items were mandatory, and participants were required to complete all items. (3) Upon completion, all returned questionnaires were reviewed individually by the researchers, and those containing errors or unclear responses were excluded. During the data collection process, to reduce social desirability bias, patients were informed that the questionnaire was anonymous and that their responses would not affect their routine care or their relationship with the nursing staff. The questionnaires were completed in a private setting (a separate room) without the presence of other patients or non-essential staff. The nursing staff who collected the data were specifically trained to maintain a neutral, non-judgmental attitude and to emphasize the importance of honest answers for research purposes. The questionnaire did not request any identifying information (name, medical record number), and sealed collection boxes were used. Nevertheless, we fully recognize that social desirability bias cannot be completely eliminated with self-report methods.

### 2.5 Statistical methods

Statistical analysis was conducted using SPSS version 23.0. For bivariate comparisons, the normality of continuous variables was assessed using the Shapiro–Wilk test, and homogeneity of variances was evaluated using Levene’s test. When the assumptions of normality and equal variances were violated, or when group sizes were severely unbalanced, nonparametric tests were used: the Mann–Whitney U test for two-group comparisons and the Kruskal–Wallis *H*-test for comparisons involving three or more groups. Normally distributed continuous variables with equal variances were compared using the independent-samples t-test or one-way ANOVA. The correlation between OHIP-14 and SESS scores was evaluated using the Pearson’s correlation coefficient. Multiple linear regression analysis was performed to identify independent factors associated with OHRQoL. Stepwise multiple linear regression (forward selection with entry criterion *p* < 0.05 and removal criterion *p* > 0.10) was used to identify independent predictors of OHIP-14 scores. Multicollinearity among predictors in the final stepwise regression model was assessed using the variance inflation factor (VIF) and tolerance. A VIF < 5 was considered acceptable. To reduce the risk of overfitting and variable selection bias, we performed internal cross-validation (leave-one-out) and bootstrap resampling (500 iterations). The stability of variable selection was assessed, and the final model was required to have a subject-to-predictor ratio >10:1. Model fit was evaluated using adjusted *R*^2^ and the overall *F*-test. A *p*-value < 0.05 was considered statistically significant.

## 3 Results

### 3.1 General characteristics of older adults receiving MHD

A total of 107 patients receiving MHD were included in this study, comprising 66 males (61.68%) and 41 females (38.32%). The age ranged from 60 to 90 years, with a mean age of 67.80 ± 6.05 years. Regarding dialysis treatment, 57 patients (53.27%) underwent hemodialysis three or more times per week, whereas 50 patients (46.73%) underwent hemodialysis fewer than three times per week. Dialysis duration was ≥3 years in 57 patients (53.27%) and <3 years in 50 patients (46.73%). A total of 52 patients (48.60%) had comorbid diabetes mellitus. Additionally, in this study cohort, the use of antihistamines and antihypertensives was relatively high, while the use of other xerostomia-inducing medications (tricyclic antidepressants, calcium channel blockers, or anticholinergic agents) was very low (≤ 3 patients per class). Therefore, these low-usage medications were not included as separate categorical variables in the regression analysis.

Laboratory and dialysis-related parameters were as follows: pre-dialysis blood urea nitrogen, 29.214 ± 4.608 mmol/L; pre-dialysis serum creatinine, 868.221 ± 120.078 μmol/L; C-reactive protein, 4.745 ± 1.687 mg/L; urea clearance index (Kt/V), 1.187 ± 0.136; pre-dialysis blood glucose, 6.756 ± 1.258 mmol/L; serum potassium, 4.758 ± 0.523 mmol/L; and serum sodium, 134.748 ± 2.256 mmol/L.

### 3.2 OHIP-14 and SESS scores of older adults receiving MHD

Among the 107 patients receiving MHD, the mean OHIP-14 score was 18.97 ± 8.00. A total of 77 patients (71.96%) had scores ≥12, whereas 30 (28.04%) had scores <12. The mean SESS score was 43.52 ± 12.98. Detailed results are presented in Table 1.

**Table 1:** Univariate analysis of factors associated with OHRQoL in older adults receiving maintenance hemodialysis (*n* = 107).

Variable	*n*	OHIP-14 (score, $\bar{x\;}$ ± s)	*t*/*F/U/H*	*p*
Sex			−0.822	0.636
Male	66	18.47 ± 8.21		
Female	41	19.78 ± 7.69		
Age (years)			0.015	0.949
60–69	69	18.07 ± 9.41		
70–79	31	19.46 ± 8.31		
≥80	7	19.65 ± 8.67		
Education level			320.512	0.054
Below senior high school	100	19.55 ± 9.21		
Senior high school and above	7	16.75 ± 7.67		
Dialysis frequency			198.053	0.006
≥3 times/week	57	11.57 ± 3.24		
<3 times/week	50	18.37 ± 11.48		
Dialysis duration (years)			15.772	<0.001
≥3	57	17.31 ± 8.15		
<3	50	12.37 ± 5.03		
Comorbid diabetes mellitus			3.522	<0.001
Yes	52	21.64 ± 7.73		
No	55	14.46 ± 5.49		
Household economic status			−0.325	0.206
Good	46	18.57 ± 7.24		
Average or poor	61	19.28 ± 8.58		
Smoking status			−0.250	0.754
Yes	49	18.45 ± 8.18		
No	58	19.41 ± 7.90		
Long-term antihypertensive medication use			2.792	0.003
Yes	58	20.90 ± 7.30		
No	49	12.69 ± 6.27		
Long-term antihistamine use			−2.502	0.486
Yes	60	17.12 ± 5.27		
No	47	16.05 ± 5.29		
Toothbrushing frequency			1.078	0.011
≥2 times/day	56	12.35 ± 3.55		
<2 times/day	51	17.21 ± 6.33		
Toothbrushing duration			350.804	0.007
≥3 min/session	52	13.00 ± 4.52		
<3 min/session	55	16.56 ± 6.20		
Mouthwash use			−3.685	0.221
Yes	11	21.91 ± 7.08		
No	96	18.64 ± 8.07		
Regular dental check-ups			−1.247	0.588
Yes	13	16.39 ± 7.00		
No	94	19.33 ± 8.10		
Tooth loss			1.870	0.051
Yes	54	20.39 ± 8.94		
No	53	17.53 ± 6.70		
SESS score			15.328	<0.001
Low	72	19.28 ± 8.37		
Moderate	20	15.41 ± 7.36		
High	15	11.03 ± 4.32		

(1) *F*, one-way analysis of variance; (2) *t*, independent samples *t*-test; (3) *U*, Mann–Whitney *U*-test; (4) *H*, Kruskal–Wallis *H*-test.

### 3.3 Univariate analysis of OHRQoL in older adults receiving MHD

Dialysis frequency, dialysis duration, comorbid diabetes mellitus, long-term use of antihypertensive medications, toothbrushing frequency, toothbrushing duration, and SESS scores were significantly associated with OHRQoL in patients receiving MHD (*p* < 0.05). Detailed results are presented in Table 1.

### 3.4 Correlation analysis between OHIP-14 scores and clinical variables

**Figure 1:**
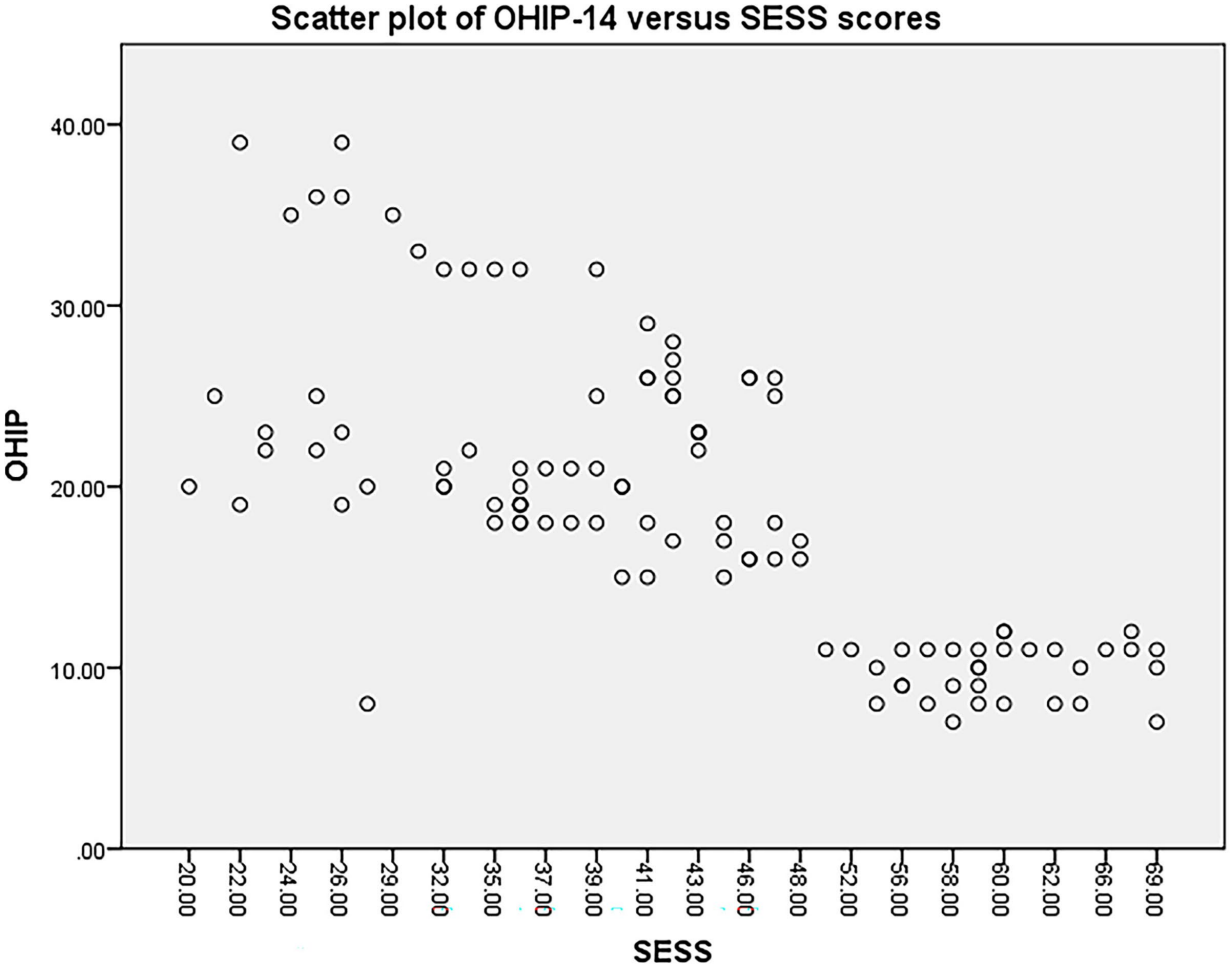
The correlation between oral self-efficacy and OHIP-14 scores.

OHIP-14 scores demonstrated a significant negative correlation with SESS scores (*r* = −0.736, *p* < 0.01, Figure 1) and urea clearance (*r* = −0.628, *p* < 0.01), and significant positive correlation with pre-dialysis blood urea nitrogen (*r* = 0.681, *p* < 0.01), pre-dialysis serum creatinine (*r* = 0.712, *p* < 0.01), and C-reactive protein (*r* = 0.703, *p* < 0.01). No statistically significant correlations were observed between OHIP-14 scores and pre-dialysis blood glucose, serum potassium, or serum sodium (*p* > 0.05). Detailed results are presented in Table 2.

**Table 2:** Pearson correlation matrix among clinical continuous variables (*n* = 107).

Variable	Pre-dialysis blood urea nitrogen	Pre-dialysis serum creatinine	Pre-dialysis serum uric acid	Blood glucose	Serum potassium	Serum sodium	C-reactive protein	Urea clearance	OHIP‑14	SESS
Pre-dialysis blood urea nitrogen (mmol/L)	1.00	0.68***	0.18	0.10	0.07	−0.09	0.52***	−0.49***	0.68***	−0.55***
Pre-dialysis serum creatinine(μmol/L)	0.68***	1.00	0.21*	0.09	0.08	−0.07	0.55***	−0.51***	0.71***	−0.58***
Pre-dialysis serum uric acid(μmol/L)	0.18	0.21*	1.00	0.05	0.11	−0.04	0.15	−0.12	0.12	−0.09
Blood glucose (mmol/L)	0.10	0.09	0.05	1.00	0.02	−0.01	0.08	−0.05	0.08	−0.06
Serum potassium(mmol/L)	0.07	0.08	0.11	0.02	1.00	0.13	0.06	−0.04	0.04	−0.03
Serum sodium(mmol/L)	−0.09	−0.07	−0.04	−0.01	0.13	1.00	−0.08	0.06	−0.06	0.05
C-reactive protein(mg/L)	0.52***	0.55***	0.15	0.08	0.06	−0.08	1.00	−0.45***	0.70***	−0.51***
Urea clearance	−0.49***	−0.51***	−0.12	−0.05	−0.04	0.06	−0.45***	1.00	−0.63***	0.48***
OHIP‑14	0.68***	0.71***	0.12	0.08	0.04	−0.06	0.70***	−0.63***	1.00	0.74***
SESS	−0.55***	−0.58***	−0.09	−0.06	−0.03	0.05	−0.51***	0.48***	−0.74***	1.00

*Correlation coefficients (*r*) are shown. *p < 0.05, **p < 0.01, ***p < 0.001 (two tailed).

### 3.5 Factors associated with OHRQoL in older adults receiving MHD

Using the OHIP-14 score as the dependent variable, variables identified as statistically significant in the univariate analysis were entered as independent variables into a multiple linear regression analysis model. Variable coding is presented in Table 3. The analysis demonstrated that dialysis frequency, dialysis duration, daily toothbrushing frequency, toothbrushing duration per session, pre-dialysis blood urea nitrogen level, pre-dialysis serum creatinine level, and the presence of comorbid diabetes mellitus were independent factors associated with OHRQoL in patients receiving MHD (*p* < 0.05). Detailed regression analysis results are presented in Table 4. In the final hierarchical regression model, age, sex, education level, C-reactive protein (CRP), urea clearance index (Kt/V), and long-term antihypertensive use were not independently associated with OHIP-14 scores (all *p* > 0.05).

**Table 3:** Coding scheme for independent variables included in the multiple linear regression analysis.

Independent variable	Coding
Dialysis frequency	≥3 times/week = 0, <3 times/week = 1
Dialysis duration	≥3 years = 0, <3 years = 1
Comorbid diabetes mellitus	Yes = 0, No = 1
Long-term antihypertensive medication use	Yes = 0, No = 1
Toothbrushing frequency	≥2 times/day = 0, <2 times/day = 1
Toothbrushing duration	≥3 min/session = 0, <3 min/session = 1
SESS	Entered as a continuous variable
Pre-dialysis blood urea nitrogen	Entered as a continuous variable
Pre-dialysis serum creatinine	Entered as a continuous variable
C-reactive protein	Entered as a continuous variable
Urea clearance	Entered as a continuous variable

**Table 4:** Stepwise multiple linear regression analysis of factors associated with OHRQoL in older adults receiving maintenance hemodialysis (*n* = 107).

Variable	Unstandardized *B* (95% CI)	SE	Standardized *β*	*t*	*p*	VIF
Constant	−3.86 (−7.62, −0.10)	1.91	–	−2.02	0.046	–
Toothbrushing frequency	−5.25 (−7.45, −3.05)	0.66	−0.10	−2.55	0.012	1.32
Pre-dialysis blood urea nitrogen	0.36 (0.13, 0.59)	0.11	0.27	3.27	0.001	2.08
Pre-dialysis serum creatinine	0.011 (0.003, 0.019)	0.004	0.16	2.75	0.007	2.15
Toothbrushing duration	−2.65 (−4.98, −0.32)	1.18	−0.16	−2.25	0.027	1.43
Dialysis frequency	−1.68 (−2.98, −0.38)	0.66	−0.10	−2.55	0.012	1.32
Dialysis duration	0.17 (0.07, 0.27)	0.05	0.11	3.40	0.001	1.45
Comorbid diabetes mellitus	1.58 (0.30, 2.86)	0.64	0.08	2.47	0.015	1.28
SESS	−0.32 (−0.45, −0.19)	0.08	−0.28	−3.88	<0.001	1.36

*R*^2^ = 0.612, adjusted *R*^2^ = 0.591, *F* (8, 98) = 19.25, *p* < 0.001. VIF, variance inflation factor; all VIF <5 indicate no problematic multicollinearity.

## 4 Discussion

### 4.1 Status of OHRQoL in older adults receiving MHD

The survey results showed that a total of 77 older adult care maintenance hemodialysis patients had OHIP-14 scores ≥12, accounting for 71.96% of the total number. These results indicate that OHRQoL is generally poor in this population and that oral health problems are more severe than those observed in the general population (16). Several factors may contribute to these findings. First, long-term hemodialysis is associated with the accumulation of metabolic toxins, which may alter the intraoral microecological environment. Second, in the context of diabetes mellitus, elevated levels of pro-inflammatory cytokines and increased insulin resistance may disrupt glucose metabolism, promoting the formation of advanced glycation end products (AGEs). These changes may alter the composition of the oral microbiota and impair periodontal microcirculation, thereby contributing to the development or progression of periodontitis. Furthermore, fluctuations in blood volume during hemodialysis may complicate blood pressure regulation, often necessitating long-term use of antihypertensive medications. Prolonged use of such medications may reduce salivary secretion, resulting in xerostomia and further compromising oral health. In addition, limited awareness of oral hygiene practices—including inadequate knowledge of toothbrushing frequency, technique, and duration—may further contribute to poor oral health outcomes. A case–control study from Iran (8) investigated OHRQoL in hemodialysis patients and reported a mean OHIP-14 score of 21.3 ± 8.1 in the case group (*n* = 140). This study is particularly relevant as it used the same OHIP-14 scale and also examined correlations with self-efficacy-related measures. Comparing our findings with this Iranian sample, the mean OHIP-14 score in our study (18.97 ± 8.00) was slightly lower, suggesting somewhat better OHRQoL in our patients. These differences may be attributable to variations in dialysis adequacy, socioeconomic status, or cultural attitudes toward oral health. Furthermore, a cross-sectional study (4) involving 120 hemodialysis patients reported a mean OHIP-14 score of 19.4 ± 7.7, which is very close to our finding. This similarity supports the argument that our results are not unique to the Chinese population but may reflect broader, potentially global, patterns of OHRQoL impairment in patients undergoing maintenance hemodialysis.

### 4.2 Factors associated with OHRQoL in older adults receiving MHD

#### 4.2.1 Oral health behaviors

Oral health behaviors represent an important factor associated with OHRQoL in patients receiving MHD, with toothbrushing frequency and duration identified as particularly influential. According to the *Oral Health Guideline for Chinese Population*, brushing at least twice daily for approximately 3 min per session is recommended as a basic standard for maintaining oral hygiene (17). Thorough tooth brushing is associated with improved oral health-related quality of life (OHRQoL) (18).

In the present study, 56 patients (52.34%) reported brushing their teeth at least twice daily, and 52 patients (48.60%) reported brushing for 3 min or longer per session. These proportions were lower than those reported by Kakudate et al. (9) in Japan and Wolfe et al. (19) in Germany. Further analysis demonstrated that patients who consistently brushed their teeth at least twice daily for a minimum of 3 min per session had significantly lower OHIP-14 scores compared with those who did not, consistent with findings by Pakpour (8). Since social desirability bias cannot be completely eliminated, the self-reported toothbrushing frequency among older adults may have been overestimated. Consequently, the actual oral hygiene status of older MHD patients may be even poorer than reported, and the protective effect of adequate toothbrushing on oral health-related quality of life (OHRQoL) may be stronger than that observed in the present study. Overall, oral health knowledge among older adults receiving MHD appears to be limited, which may be associated with insufficient emphasis on oral health by healthcare providers (17). In routine clinical practice, oral examinations and oral health education are not routinely integrated into the management of patients receiving MHD. Therefore, enhanced awareness among healthcare providers and the integration of dental professionals into multidisciplinary care are warranted. Targeted interventions—such as visual demonstrations using dental models, development of illustrated educational materials, and facilitation of patient experience-sharing sessions—may help improve oral health awareness and promote the adoption and maintenance of appropriate oral health behaviors among older adults (20).

#### 4.2.2 Oral self-efficacy in older adults receiving MHD

In this study, the oral self-efficacy of older adult care maintenance hemodialysis patients was assessed. A total of 72 patients (67.29% of the total sample) exhibited low-level self-efficacy, indicating an overall low level of oral self-efficacy in this population. A significant negative correlation was observed between SESS and OHIP-14 scores (*r* = −0.736, *p* < 0.01), indicating that higher oral self-efficacy is associated with lower oral health impact scores and improved OHRQoL. A validation study (19) of the Oral Self-efficacy Scale (SESS) in patients with periodontal disease reported mean SESS scores ranging from 50 to 55 in the general adult population. In contrast, our patients exhibited much lower SESS scores (43.52 ± 12.98), which we interpret as indicating particularly low oral self-efficacy in the older MHD population compared with general dental patients in Japan. This finding underscores the need to enhance individuals’ confidence in their own oral health management capabilities and to foster a more proactive attitude toward addressing oral health issues (21). Based on these findings, the assessment and enhancement of oral self-efficacy should be incorporated into health education programs for patients receiving MHD. In addition, extending health educational initiatives to family members may provide additional support, reinforce self-efficacy, and promote the adoption and maintenance of appropriate oral health behaviors among older adults, thereby contributing to improvements in OHRQoL.

#### 4.2.3 Patient-related characteristics in older adults receiving MHD

##### 4.2.3.1 Dialysis frequency

Previous studies have demonstrated that patients receiving hemodialysis three or more times per week exhibit lower OHIP-14 scores than those receiving dialysis fewer than three times per week, indicating that lower dialysis frequency is associated with poorer OHRQoL. This association may be attributable to the accumulation of uremic toxins resulting from inadequate dialysis, which may reduce salivary flow rate and buffering capacity, thereby impairing the self-cleansing ability of the oral cavity (1).

##### 4.2.3.2 Dialysis duration

Patients with a dialysis duration exceeding 3 years demonstrated significantly higher OHIP-14 scores than those with a duration of 3 years or less. With increasing dialysis duration, oral hygiene status—reflected by indices such as the calculus index and debris index—demonstrated progressive deterioration, accompanied by an increased prevalence and severity of periodontal disease. We compared the effects of dialysis frequency and duration on OHRQoL with international findings. Our observation that longer dialysis vintage is associated with worse OHRQoL is consistent with Cengiz (21) in Turkey, who reported a progressive deterioration of oral hygiene indices as dialysis duration increased. The underlying mechanisms may be associated with impaired immune function in the uremic state, which increases susceptibility to subgingival colonization and proliferation of Gram-negative bacteria. In addition, fluid intake restrictions may exacerbate xerostomia, alter the physicochemical properties of saliva, and further compromise oral defense mechanisms (22).

##### 4.2.3.3 Comorbid diabetes mellitus

Patients receiving MHD with comorbid diabetes mellitus exhibited higher OHIP-14 scores than those without diabetes mellitus, indicating that diabetes mellitus is an important factor associated with OHRQoL in this population. Diabetes mellitus is associated with reduced salivary secretion and decreased salivary enzymatic activity, resulting in oral mucosal dryness and impaired self-cleansing capacity. Diabetes is associated with faster alveolar bone resorption and the occurrence and progression of attachment loss (21). These effects may be mediated by alterations in host immune function, which influence oral microbial balance and local inflammatory responses. Specifically, diabetes may impair leukocyte function, enhance monocyte–macrophage reactivity, activate the protein kinase C (PKC) signaling pathway, and promote the formation and accumulation of advanced glycation end products (AGEs). These factors collectively contribute to alterations in periodontal tissue and the progression of periodontal disease (23).

Based on these findings, enhanced communication between healthcare providers and patients receiving MHD is warranted to facilitate the development of individualized, evidence-based management strategies. For patients with insufficient dialysis frequency, increasing the frequency of dialysis sessions may help reduce systemic toxin burden. For those with comorbid diabetes mellitus, optimization of glycemic control, including regular blood glucose monitoring and timely intervention, may help maintain blood glucose within target ranges and mitigate the onset and progression of oral complications.

#### 4.2.4 Polypharmacy

We also recognize that other medications frequently prescribed to the older adults, such as tricyclic antidepressants, calcium channel blockers, and anticholinergic agents, are known to induce xerostomia and may have an impact on Oral Health-Related Quality of Life (OHRQoL). In our cohort, the prevalence of these medications was extremely low (≤3 patients per class), which precluded separate analysis. Nevertheless, polypharmacy involving these agents remains a potential confounding factor. Future studies with larger sample sizes should specifically collect and analyze data on these medication classes to gain a better understanding of their contribution to OHRQoL in older patients with Maintenance Hemodialysis (MHD).

#### 4.2.5 Biochemical indicators in older adults receiving MHD

The findings of this study indicate that pre-dialysis blood urea nitrogen and pre-dialysis serum creatinine levels are associated with OHRQoL, likely through their effects on the oral microenvironment. Furthermore, high levels of toxins are associated with the patient’s oral’ ammoniacal odor,’ which may subsequently impair their social functioning and quality of life (24). Adequate dialysis, typically defined as a weekly duration of at least 12 h, may help maintain appropriate metabolic control. Although reducing dialysis frequency may alleviate financial burden and improve treatment adherence, it may also result in inadequate dialysis, thereby adversely affecting pre-dialysis blood urea nitrogen and serum creatinine levels.

We now briefly interpret two notable non-significant findings:

CRP: Although CRP showed a moderate positive correlation with OHIP-14 in univariate analysis (*r* = 0.703, *p* < 0.001, as reported in section 2.4), it became non-significant in the multivariate model. We suggest that this may be because CRP’s effect on OHRQoL is largely mediated by dialysis adequacy (Kt/V) and nutritional status (albumin, BUN), which were already included in the model. Alternatively, the narrow range of CRP values in our stable outpatient cohort (mean 4.75 ± 1.69 mg/L) might have limited its discriminatory power. We cite one relevant reference to support the known link between inflammation and oral health in dialysis patients (11), while acknowledging that our study could not isolate an independent effect. Kt/V: The urea clearance index was not significantly associated with OHIP-14 (*p* = 0.12). A possible explanation is that Kt/V in our sample was relatively high (mean 1.187 ± 0.136) with limited variability, reflecting good dialysis adequacy in most patients. Moreover, Kt/V is a global measure of small-molecule clearance, whereas pre-dialysis BUN and creatinine––which were significant––more directly reflect the peak concentrations of uremic toxins that affect oral mucosal health and breath odor. This interpretation is consistent with previous literature (1). Long-term antihypertensive use: this variable was significant in univariate analysis (Table 1) but not in the multivariate model. The loss of significance may be due to its overlap with dialysis duration and diabetes, both of which were independently associated with OHRQoL (25). Furthermore, the specific types of antihypertensives varied (e.g., CCBs, ARBs, beta-blockers), and we did not have sufficient power to analyze each class separately (26).

Several limitations ought to be recognized. Firstly, the cross-sectional design precludes any causal inference. For instance, although a strong negative correlation between oral self-efficacy and Oral Health-Related Quality of Life (OHRQoL) was observed, the directionality remains ambiguous––poor OHRQoL might also impair self-efficacy. Secondly, convenience sampling from a single tertiary hospital gives rise to selection bias. Patients in our hospital may have more advanced comorbidities and better access to specialized care, which restricts the generalizability to older MHD patients in community-based or rural dialysis settings. We acknowledge that an *a priori* power calculation was not performed due to the lack of prior data; however, the post-hoc analysis supports the adequacy of the sample. Third, stepwise regression was used for variable selection, which is known to carry risks of overfitting and biased coefficient estimates. Although we implemented cross-validation and bootstrapping to mitigate these issues, the results should be considered hypothesis-generating. Therefore, our findings should be regarded as hypothesis-generating, and future longitudinal, multi-center studies are necessary to confirm the observed associations and to evaluate causality.

## 5 Conclusion

The findings of this study indicate that OHRQoL is generally low among individuals receiving MHD. Multivariate analysis identified dialysis frequency, dialysis duration, daily toothbrushing frequency, toothbrushing duration per session, pre-dialysis blood urea nitrogen level, pre-dialysis serum creatinine level, comorbid diabetes mellitus, and oral self-efficacy as key factors associated with OHRQoL in this population.

## Data Availability

The original contributions presented in the study are included in the article/supplementary material, further inquiries can be directed to the corresponding authors.
